# Development of Machine Learning-Based Sub-Models for Predicting Net Protein Requirements in Lactating Dairy Cows

**DOI:** 10.3390/ani15142127

**Published:** 2025-07-18

**Authors:** Mingyung Lee, Dong Hyeon Kim, Seongwon Seo, Luis O. Tedeschi

**Affiliations:** 1 Department of Animal Science, Texas A&M University, College Station, TX 77843-2471, USA; mingyung@tamu.edu; 2 Dairy Science Division, National Institute of Animal Science, Rural Development Administration, Cheonan 31000, Republic of Korea; kimdh3465@korea.kr; 3 Division of Animal and Dairy Sciences, Chungnam National University, Daejeon 34134, Republic of Korea; swseo@cnu.ac.kr

**Keywords:** lactating Holstein cows, net protein for lactation, net protein for maintenance, random forest regression, support vector regression

## Abstract

A reliable estimation of protein requirements in lactating dairy cows is necessary for formulating nutritionally adequate diets, improving feed efficiency, and minimizing nitrogen excretion. This study aimed to develop machine learning-based models to predict net protein requirements for maintenance (NPm) and lactation (NPl) using random forest regression (RFR) and support vector regression (SVR). A total of 1779 observations were assembled from 436 peer-reviewed publications and open-access databases. Predictor variables included farm-ready variables such as milk yield, dry matter intake, days in milk, body weight, and dietary crude protein content. NPm was estimated based on the National Academies of Sciences, Engineering, and Medicine (NASEM, 2021) equations, while NPl was derived from milk true protein yield. The model adequacy was evaluated using 10-fold cross-validation. The RFR model demonstrated higher predictive performance than SVR for both NPm (R^2^ = 0.82, RMSEP = 22.38 g/d, CCC = 0.89) and NPl (R^2^ = 0.82, RMSEP = 95.17 g/d, CCC = 0.89), reflecting its capacity to model the rule-based nature of the NASEM equations. These findings suggest that RFR may provide a valuable approach for estimating protein requirements with fewer input variables. Further research should focus on validating these models under field conditions and exploring hybrid modeling frameworks that integrate mechanistic and machine learning approaches.

## Simple Summary

Accurately estimating the protein requirements of lactating dairy cows is crucial for enhancing nutrient utilization and promoting sustainable milk production. This study explored the application of machine learning (ML) techniques to predict two components of protein requirement—net protein for maintenance (NPm) and net protein for lactation (NPl)—utilizing a dataset compiled from published experimental studies on lactating Holstein cows. Random forest regression (RFR) and support vector regression (SVR) algorithms were trained to approximate target values calculated using the NASEM (2021) equations. Among the two approaches, the RFR model consistently outperformed SVR in terms of predictive accuracy, explaining over 80 percent of the variation in both NPm and NPl. These findings demonstrate the potential of ML models to serve as surrogate approximators of mechanistic outputs, offering computational efficiency and practical applicability in precision feeding programs. Further research is warranted to validate these models under field conditions and to explore their integration into hybrid modeling frameworks.

## 1. Introduction

Accurately quantifying nutrient requirements is fundamental to the nutritional management of lactating dairy cows, as it directly influences milk production, animal health, economic viability, and the long-term sustainability of dairy operations [1,2,3]. Among these nutrients, protein plays a pivotal role not only as the principal substrate for milk protein synthesis but also as a critical component supporting a range of metabolic and physiological processes in lactating cows [4,5]. Unlike metabolizable protein, which denotes the quantity of absorbable amino acids supplied to the small intestine, net protein requirements (NP) represent the actual protein demands of the animal for maintenance (NPm) and lactation (NPl), adjusted for metabolic losses and the efficiency of utilization [6,7]. Reliable estimates of NP are essential for aligning nutrient supply with physiological demand, given that overestimation can lead to inefficient nitrogen utilization and increased environmental emissions such as ammonia volatilization and nitrate leaching. In contrast, underestimation may compromise milk yield (MY) and animal welfare [8,9].

To address these challenges, NP requirements have traditionally been estimated using mechanistic feeding models, including those proposed by the NRC (2001; [6]), its recent update by the NASEM (2021; [7]), and many nutrition models for ruminant animals based on the Cornell Net Carbohydrate and Protein System framework such as the Ruminant Nutrition System [10,11]. These models employ fixed-form biological equations based on variables such as feed composition, body weight, and milk production. While these equations are grounded in well-established physiological principles, their applicability may be constrained in heterogeneous production environments or under dynamically changing farm conditions [12,13,14].

Recent advances in machine learning (ML) offer a promising alternative to traditional modeling approaches by enabling data-driven characterization of complex and nonlinear biological systems [15,16,17]. ML algorithms, such as random forest and support vector machine algorithms, are particularly effective when applied to high-dimensional datasets and are capable of identifying latent patterns and variable interactions that may be overlooked by conventional models [18,19]. In dairy science, ML has been applied to a variety of predictive tasks, including MY, feed intake, and metabolic disorders [20,21]. However, its application for estimating nutrient requirements, particularly as surrogate models that replicate outputs of mechanistic systems, remains relatively underinvestigated.

The objective of the present study was to develop machine learning-based models for predicting NP requirements for maintenance and lactation in lactating dairy cows. The target values used to train the ML models were not obtained from direct empirical measurements, but were derived from the NASEM (2021) equations because direct measurements of net protein requirements are not readily available. As such, these models are intended to serve as computationally efficient approximators of NASEM predictions, like a meta-modeling approach, rather than replacements for the underlying biological framework. We hypothesized that machine learning models can approximate NASEM (2021)-based predictions of protein requirements using readily available input variables. This study builds upon our previous work, which developed ML models to predict metabolizable protein supply components, namely rumen-undegradable protein (RUP) and duodenal microbial nitrogen (MicN), using the same comprehensive database [22]. While the earlier study addressed the supply side of protein nutrition, this study focuses on the requirement side. These ML-based sub-models are expected to enhance usability, reduce implementation complexity, and support real-time decision-making in precision feeding programs. Furthermore, this work provides a foundation for the development of hybrid intelligent mechanistic models (HIMM; [17,23,24]), which aim to combine mechanistic insight with data-driven flexibility to improve the accuracy and adaptability of dairy nutritional management.

## 2. Materials and Methods

### 2.1. Database Construction and Dataset Extraction

The dataset used in this study was identical to that developed and described by Lee et al. [22]. Briefly, it was constructed by integrating two publicly available and peer-reviewed databases, both of which contain observations exclusively from lactating Holstein cows. The final combined dataset included 1779 observations extracted from 436 peer-reviewed publications. The first component was obtained from 319 articles published between 2016 and 2020 in the Animal Nutrition section of the Journal of Dairy Science, which contributed 1326 observations and had been previously compiled by Jeon et al. [25]. The second component was obtained from the National Animal Nutrition Program (https://animalnutrition.org; accessed on 17 October 2023), comprising 453 observations from 117 articles published between 1979 and 2017. The dataset represents a wide range of geographical and production contexts, including studies conducted in North America, Europe, Asia, Africa, and Latin America, and covers various feeding systems such as total mixed ration and pasture-based operations. To ensure the integrity of the dataset, all records were thoroughly screened to eliminate duplication across the two sources. A full description of the database construction process, along with summary statistics, was provided by Lee et al. [22].

### 2.2. Variable Selection and Calculations

For NPm (g/d), the reference values were calculated as the sum of scurf protein, endogenous urinary protein, and metabolic fecal protein based on equations proposed by NASEM (2021). The NPm (g/d) was estimated as follows:

NPm (g/d) = NP_scurf_ + NP_endogenous urinary excretion_ + NP_metabolic fecal protein_ (1)

The scurf protein requirement was calculated as follows ([7]):

NP_scurf_ (g/d) = 0.20 × BW^0.60^ × (TP/CP) (2)

where BW represents body weight in kilograms, TP is true protein, CP is crude protein, and the TP/CP ratio of scurf is 0.85. 

The endogenous urinary protein requirement was calculated using the following equation ([7]):

NP_endogenous urinary excretion_ (g/d) = 53 × 6.25 × BW × 0.001 (3)

where 53 (mg N/kg BW) is the endogenous urinary nitrogen excretion per kilogram of BW, 6.25 is the standard conversion factor from nitrogen to crude protein, and the TP/CP ratio for endogenous urinary protein is 1.0.

Metabolic fecal protein was estimated as follows ([7]):

NP_metabolic fecal protein_ (g/d) = [(11.62 + 0.134 × NDF) × DMI] × 0.73 (4)

where NDF is the percentage of neutral detergent fiber on a dry matter basis, and DMI is the daily dry matter intake (kg). The TP/CP ratio for metabolic fecal protein is 0.73.

The reference NPl (g/d) value was estimated based on the milk true protein content using the following equation:

NPl (g/d) = MY × Milk CP × 0.951 (1000/100)(5)

where MY is milk yield (kg), Milk CP represents the crude protein concentration in milk (%), and the TP/CP ratio for milk is 0.951. Milk CP values were directly obtained from the datasets and were not calculated from nitrogen; hence, no N-to-CP conversion factor (e.g., 6.34) was applied.

To prevent circular reasoning in model development, variables directly used in the calculation of NPm and NPl values based on the NASEM (2021) equations were excluded from the candidate predictor set. Instead, candidate variables were selected based on biological relevance and availability under typical field conditions. These included animal-related factors such as days in milk (DIM), parity, BW (kg), DMI (kg/d), and MY (kg/d), as well as dietary components expressed as a percentage of dry matter (% DM), including dry matter (DM, % as-fed), organic matter (OM), CP, neutral detergent fiber (NDF), acid detergent fiber (ADF), non-starch carbohydrate (NSC), fat, ash, and starch. Although BW, MY, DMI, and NDF are included in the NASEM equations, each variable was retained as a model input only when not directly used in the calculation of the corresponding target variable. Specifically, BW, DMI, and NDF were excluded from the NPm prediction model, and MY was excluded from the NPl prediction model. Additionally, variables such as milk composition, total tract digestibility, rumen characteristics, and post-digestion outcomes were also excluded as these variables are not always available at the farm level. Separate datasets were constructed for NPm and NPl prediction, each comprising the target variable and its respective candidate explanatory variables. Descriptive statistics for these datasets are provided in Table 1 and Table 2.

### 2.3. Model Development and Evaluation

Variables with insufficient data were excluded from the dataset before model development for predicting NPm and NPl. The resulting dataset was partitioned into training (80%) and test (20%) sets. The training set was further split into a sub-training set (80%) and a validation set (20%) for the purpose of selecting the optimal combination of input variables and hyperparameters. Model selection was based on performance in the validation set, evaluated using the coefficient of determination (R^2^), root mean square error of prediction (RMSEP), and concordance correlation coefficient (CCC), with an emphasis on achieving both high precision and accuracy. The optimal model was subsequently retrained using the entire training set and evaluated on the independent test set. A new dataset containing only the input variables selected for the final model was then extracted from the original dataset, and a 10-fold cross-validation was conducted to assess the model’s robustness and generalizability. An overview of the dataset refinement and modeling procedures was presented by Lee et al. [22].

All statistical analyses and modeling procedures were performed using R software (version 4.4.2; [26]). To predict NPm and NPl, both random forest regression (RFR) and support vector regression (SVR) algorithms were applied. SVR, an extension of the support vector machine algorithm, was implemented using a Gaussian radial basis function kernel, which includes two key hyperparameters: gamma and cost [27,28]. These hyperparameters were optimized via grid search within a 10-fold cross-validation framework using the tune function in the e1071 package [29] in R. Random forest algorithm is a widely used ensemble learning method suitable for both classification and regression tasks [18], and is largely employed in the animal science literature [30]. Its hyperparameters, namely the number of trees (ntree) and the number of variables randomly selected at each split (mtry), were fine-tuned through grid search. Modeling was performed using the randomForest package [31] in R. Model adequacy between predicted values and reference values calculated from the NASEM (2021) equations was evaluated using several statistical metrics as described by Tedeschi [32], including the R^2^, RMSEP, and CCC. The strength of agreement based on CCC was interpreted using the categories proposed by Hinkle et al. [33]: negligible (0.00–0.30), low (0.30–0.50), moderate (0.50–0.70), high (0.70–0.90), and very high (0.90–1.00). Residual analyses were conducted to assess both mean and slope biases. Statistical significance was declared at *p* < 0.05, and trends were considered for 0.05 ≤ *p* < 0.10.

## 3. Results

### 3.1. NPm Prediction

**Figure 1:**
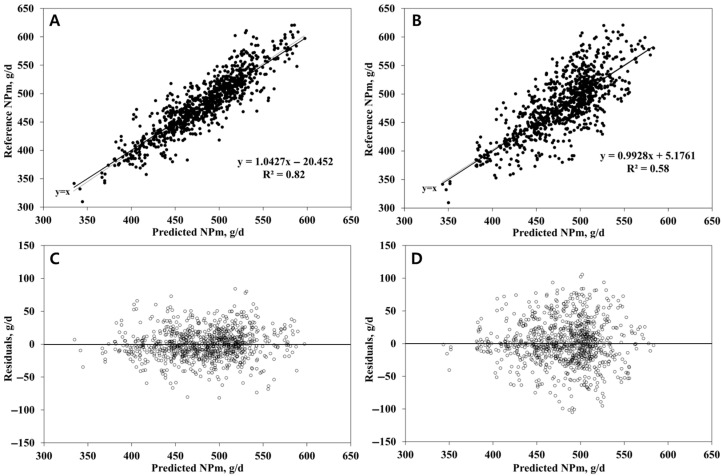
Plots of the reference (upper) and residual values (reference minus predicted values, lower) versus the model-predicted net protein requirement for maintenance (g/d), based on predictions (*n* = 1288) from 10-fold cross-validation. (**A**,**C**) are plots of a model developed with a random forest regression algorithm. (**B**,**D**) are plots of a model developed with a support vector regression algorithm.

In the dataset constructed for NPm prediction, variables with insufficient observations were excluded to improve model reliability. Specifically, dietary OM, fat, ash, NSC, and starch were removed if the number of observations was fewer than 100 (i.e., <20% of the dataset) or if their co-occurrence with other predictors was insufficient. After filtering, 288 treatment means were retained. Of these, 80% (*n* = 231) were used for model development, and the remaining 20% (*n* = 57) were reserved as a hold-out test set. The final input variables, which were MY, DIM, and parity, were selected based on validation performance. The RFR model, tuned with 150 trees and 3 variables per split, outperformed the SVR model (cost = 5, gamma = 1) in predictive ability. As summarized in Table 3, RFR achieved 18% higher R^2^ than SVR (0.77 vs. 0.65) and reduced prediction error by approximately 6 g/d (RMSEP: 23.35 vs. 29.35 g/d). Agreement with reference values, as reflected by CCC, was also higher for RFR (0.87 vs. 0.80). In both RFR and SVR models, mean and slope biases were statistically non-significant (*p* > 0.10), while random bias accounted for over 95% of the RMSEP. Model generalizability was evaluated using 10-fold cross-validation on the full dataset (*n* = 1288) (Table 3). The RFR model maintained its advantage, yielding 41% higher R^2^ and 11 g/d lower RMSEP compared to SVR (RFR: R^2^ = 0.82, RMSEP = 22.38 g/d; SVR: R^2^ = 0.58, RMSEP = 33.34 g/d). CCC values similarly favored RFR (0.89 vs. 0.73). In both RFR and SVR models, random bias accounted for most of the prediction errors, contributing more than 95% of the RMSEP across all validation methods. By contrast, mean and slope bias represented only minor proportions of the error structure. These findings are graphically supported by Figure 1. Variable importance was computed across folds, normalized to sum to one, and averaged. Among the input variables, MY was identified as the most influential predictor for NPm, with a relative importance score of 0.515 ± 0.006, followed by DIM (0.374 ± 0.009) and parity (0.111 ± 0.010).

### 3.2. NPl Prediction

**Figure 2:**
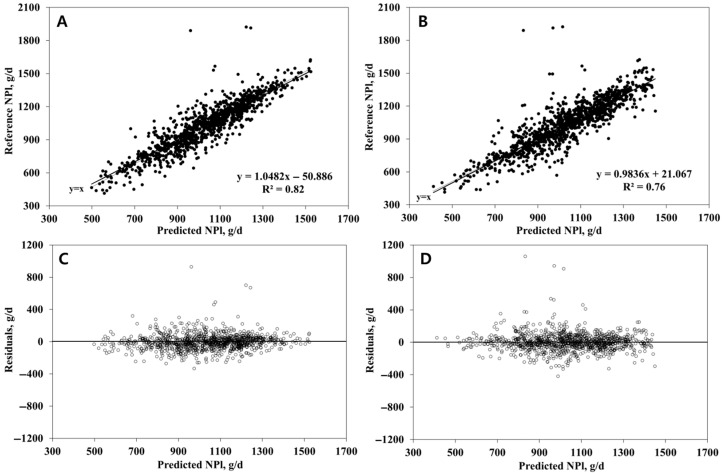
Plots of the reference (upper) and residual values (reference minus predicted values, lower) versus the model-predicted net protein requirement for lactation (g/d), based on predictions (*n* = 668) from 10-fold cross-validation. (**A**,**C**) are plots of a model developed with a random forest regression algorithm. (**B**,**D**) are plots of a model developed with a support vector regression algorithm.

The same exclusion criteria were applied to the NPl dataset. After filtering, 668 treatment means remained, with 80% (*n* = 534) used for model development and 20% (*n* = 134) for evaluation. The final selected input variables were DMI, DIM, BW, and dietary CP concentration. The RFR model was set up with ntree = 150 and mtry = 3, and the SVR model used cost = 1 and gamma = 1. As shown in Table 4, both models performed comparably on the test set, with R^2^ values differing by only 0.01 (0.79 vs. 0.78) and RMSEP values differing by less than 1 g/d (90.45 vs. 91.36 g/d). CCC was identical (0.88), and mean and slope biases were minimal and statistically insignificant (*p* > 0.10), with random error accounting for over 99% of the total prediction error. In the 10-fold cross-validation (*n* = 668), RFR achieved higher predictive accuracy, with 8% higher R^2^ and a 13.9 g/d reduction in RMSEP relative to SVR (RFR: R^2^ = 0.82, RMSEP = 95.17 g/d; SVR: R^2^ = 0.76, RMSEP = 109.06 g/d) (Table 4 and Figure 2). CCC was also slightly higher for RFR (0.89 vs. 0.86), and random error contributed more than 98% to the total prediction error in both models. As in the NPl prediction, where variable importance was averaged across folds, DMI emerged as the most dominant predictor (0.526 ± 0.009), followed by DIM (0.236 ± 0.007), BW (0.151 ± 0.004), and CP (0.086 ± 0.004).

## 4. Discussion

Understanding the NP requirements of lactating dairy cows is a critical component of formulating diets that meet physiological needs while minimizing nitrogen waste [8,34]. Mechanistic models, such as those provided by NASEM (2021), offer structured estimations based on biological principles; however, their practical use can be constrained by computational complexity, rigid input requirements, and limited adaptability to diverse farm conditions. These limitations often arise from fixed equations that do not account for nonlinear interactions among intake, metabolism, and production variables. In response to these challenges, the present study developed machine learning-based sub-models to approximate NASEM-predicted NP requirements using a limited set of biologically meaningful and field-accessible variables. This approach aims to enhance model usability in commercial settings and support more flexible, data-driven decision-making in precision feeding systems.

In the present study, BW was intentionally excluded as an input variable for predicting NPm. Although BW has traditionally been considered the primary determinant in mechanistic models, such as those by NASEM (2021), its removal was aimed at avoiding circular reasoning and exploring the predictive utility of alternative physiological indicators. Variables such as MY, DIM, and parity showed high predictive performance, which may be attributed to their biological relevance as well as potential indirect associations with BW [6,7]. In previous studies, these variables have been used as predictors of body weight [35,36]. However, rather than simply serving as substitutes for body size, these variables may provide more dynamic insights into metabolic activity and stage-specific protein turnover. For instance, MY can reflect the energetic burden of milk synthesis, particularly under negative energy balance during early lactation, when gluconeogenesis and tissue mobilization are intensified. DIM, in turn, serves as a temporal marker of physiological adjustments occurring throughout lactation [37,38].

The observed prominence of MY in the NPm model aligns with these physiological interpretations. Specifically, MY contributed over 50% to variable importance, approximately five times more than parity. This suggests a notable association between productive performance and maintenance protein requirements. Similar relationships have been described in nitrogen balance studies, where MY has been positively associated with both nitrogen output and metabolic protein requirements [8,39]. Furthermore, recent factorial models have emphasized the role of MY in metabolizable protein estimation [40], and data-driven approaches have identified MY as a key predictor of nitrogen excretion in dairy cows [41]. These converging lines of evidence indicate that the predictive prominence of MY in the current model not only reflects statistical structure but also captures fundamental biological processes related to protein maintenance needs.

In contrast, DMI emerged as the most dominant predictor of NPl, followed by DIM, BW, and dietary CP. This pattern is consistent with nutritional mechanisms where nutrient intake and lactation stage directly influence amino acid availability for milk protein synthesis [42,43]. Greater dry matter intake typically increases metabolizable protein supply, thereby enhancing amino acid availability for milk protein synthesis [44]. This physiological linkage supports the strong predictive role of DMI observed in the model. DMI, being a highly dynamic and variable indicator, fluctuates in response to physiological demands, feed availability, and lactation stage [45,46,47]. This makes it a more immediate and informative signal of nutrient supply and metabolic activity, naturally giving it greater influence on protein-output predictions. Similarly, DIM is important in predictive models as it reflects various complex physiological changes across lactation stages [48,49,50]. The combination of DMI and DIM in our model effectively captured both short-term nutritional input and longer-term physiological adaptation. While BW was included, its contribution was relatively modest, likely due to its more static nature compared to dynamic indicators, such as DMI [51,52,53]. Nevertheless, BW provides essential baseline information on the cow’s metabolic scale and protein reserves, offering auxiliary information that enhances prediction robustness. The relatively lower importance of dietary CP may reflect inter-animal variation in nitrogen use efficiency, influenced by factors such as microbial protein synthesis, energy–protein synchronization, and nutrient digestibility [8,54]. Although crude protein is commonly used to estimate nitrogen intake, it does not capture protein digestibility or amino acid composition, which ultimately determine the availability of metabolizable protein for milk synthesis. Nutritional models such as NASEM (2021), which form the basis for our target variables, address these limitations by partitioning dietary protein into degradable and undegradable fractions and explicitly modeling the flows of metabolizable protein and amino acids. However, such detailed inputs are rarely available in routine farm data. In this context, dietary CP was retained in our model as a practical and accessible proxy that enables the machine learning algorithm to capture important aspects of protein nutrition using commonly measured variables. Accordingly, while CP played a less dominant role than DMI or DIM, its inclusion contributed to the comprehensive explanatory power of the model by representing general nitrogen supply. Together, the four selected variables (DMI, DIM, BW, and CP) provided a biologically relevant and complementary set of predictors that supported the robustness and accuracy of the NPl model.

In the present study, the RFR model exhibited predictive accuracy that was consistently comparable to or exceeded that of the SVR model for both NPm and NPl predictions. This outcome presents a notable contrast to our previous findings [22], where the optimal model depended on the specific prediction target. In that study, RFR was superior for estimating RUP, while SVR was significantly more effective for predicting MicN. This divergence between the two studies suggests that the relative performance of these algorithms may be dependent on the intrinsic nature of the target variable. The consistent advantage of the RFR model in the current study could be attributed to the nature of the prediction targets. For this work, the machine learning models were trained to approximate NPm and NPl values that were calculated using the factorial and empirically informed equations of the NASEM (2021) model. The ensemble structure of the RFR algorithm, which partitions data based on conditional rules, appears particularly well-suited for modeling a system defined by a set of fixed equations and interacting components [18,55]. Similar strengths in handling complex interactions have been observed for RFR in other animal science fields [56,57].

Conversely, the better performance of SVR for MicN prediction in our previous study likely reflects a different underlying principle. The synthesis of microbial nitrogen is a fundamental biological process that may follow a smoother and more continuous global function in response to nutrient availability. The SVR algorithm may be better designed to capture such global trends with kernel transformations and global optimization, which can be particularly beneficial for problems involving smoother underlying functions [28,58]. There have been previous studies where SVR has demonstrated good performance in modeling complex systems in the dairy industry. For example, it has been successfully used to predict farm-level electricity consumption [59] and effectively utilized to quantify biological markers such as the somatic cell count in raw milk [60]. This interpretation is further supported by the observation in our previous study that the SVR model for MicN exhibited no significant predictive bias [22], whereas the RFR model did. Taken together, these two studies appear to provide a clear illustration of a model–problem-fit principle. When the task is to approximate a rule-based system, as in the current study, the RFR algorithm seems to be highly effective. When the task is to model a smoother biological process, such as MicN synthesis, the SVR algorithm can be more suitable. These findings are consistent with the broader literature supporting the use of tree-based models in dairy applications. For instance, a systematic review reported that decision tree-based methods were the most frequently applied in dairy production [21]. This suggests that the selection of a machine learning algorithm can be guided by the underlying structure of the problem, enabling the development of robust and flexible tools for applications in dairy nutrition modeling.

The sub-models developed in this study were designed to function as computationally efficient approximators of nutrient requirements as defined by the NASEM (2021) framework rather than to directly predict empirically measured values. This approach offers practical advantages, including compatibility with existing nutrient systems and ease of implementation in precision feeding environments. By using routinely collected variables such as MY, DIM, and DMI, the models operate with low computational demands, making them suitable for real-time dietary decision support. This feature is particularly valuable in diverse production environments where detailed input data required by mechanistic models may be limited. By relying on routinely available variables, the models offer a practical balance between biological relevance and field-level applicability. Moreover, their low computational demands enhance potential integration into real-time monitoring or decision-support tools across various farm settings. More accurate estimates of protein needs may help reduce overfeeding and subsequent nitrogen excretion, thereby contributing to sustainability goals in dairy production. However, important limitations must be acknowledged. Since the models were trained in outputs derived from mechanistic equations, they may inherit any structural biases or simplifications inherent to the NASEM framework. Furthermore, the models have not yet been subjected to external validation, meaning their performance across diverse breeds, management systems, and environmental conditions remains untested. Therefore, the immediate next step is to empirically validate these models using independent, farm-level datasets that include measured nitrogen balance data. While previous research has shown that machine learning models of nitrogen excretion can outperform traditional linear approaches [40], which lends support to the potential of our models, direct validation is essential to confirm their real-world accuracy and robustness.

Ultimately, the development of these models aligns with the broader objective, as discussed in our previous work [22], of advancing toward HIMM. This framework seeks to integrate the biological foundations of mechanistic models with the adaptive, data-driven power of machine learning. The RUP and MicN models from our prior study demonstrate how ML can refine predictions on the supply side of metabolizable protein. Complementarily, the NPm and NPl models from the present study illustrate how ML can create efficient surrogate models for the requirements side. A fully realized HIMM could integrate both types of components, using real-time data to create adaptive, individualized feeding strategies that enhance both productivity and environmental sustainability.

## 5. Conclusions

In this study, we hypothesized that machine learning models could approximate NASEM (2021)-based predictions of net protein requirements using readily available input variables. The results support this hypothesis: both NPm and NPl models, trained with limited field-accessible data from lactating Holstein dairy cows, achieved high agreement with NASEM-calculated reference values. Specifically, the RFR model explained approximately 80 percent of the variation in both outcomes, with R^2^ values of 0.82 for NPm and NPl based on 10-fold cross-validation. These findings confirm that ML algorithms, particularly tree-based methods, can serve as effective surrogate approximators of mechanistic nutrient models. Furthermore, the strong performance of models based on dynamic physiological and intake-related variables (e.g., MY and DMI) highlights their practical utility in real-time nutritional decision-making. While these models do not replace mechanistic frameworks, they offer a low-complexity tool compatible with on-farm applications. Future work should validate the models with empirical nitrogen balance data and explore their integration into HIMM to enhance flexibility and adaptability in dairy nutrition management.

## Figures and Tables

**Table 1:** Descriptive statistics of the dataset for net protein requirement for maintenance modeling.

Variables	*n*	Mean	Median	Min	Max
No. of articles	323				
No. of treatments	1288				
No. of animals per study	15,176	47.0	4.0	384.0	45.15
No. of animals per treatment	15,176	11.8	2.0	52.0	9.10
Animal information					
Days in milk (day)	1093	105.5	1.0	323.0	59.50
Parity	1084	1.8	1.0	2.0	0.28
Body weight (kg)	1288	635.1	476.0	861.9	58.56
Dry matter intake (kg/d)	1288	22.4	11.3	32.0	3.70
Milk yield (kg/d)	1251	34.3	10.2	58.5	8.17
Dietary chemical composition (% DM)					
Dry matter (% as-fed)	767	53.9	13.5	96.3	12.17
Organic matter	787	92.3	46.1	98.9	2.73
Crude protein	1280	16.6	9.3	29.6	1.96
Neutral detergent fiber	1288	32.8	17.6	60.8	5.03
Acidic detergent fiber	951	20.4	8.8	39.7	3.78
Fat	652	3.9	0.3	8.9	1.22
Ash	691	7.4	1.1	16.5	1.61
Non-starch carbohydrate	527	39.4	16.8	51.2	5.52
Starch	765	24.5	0.2	47.6	6.64
Net protein requirements for maintenance (g/d)	1288	480.3	309.7	621.1	52.24

**Table 2:** Descriptive statistics of the dataset for net protein requirement for lactation modeling.

Variables	*n*	Mean	Median	Min	Max
No. of articles	332				
No. of treatments	1584				
No. of animals per study	18,931	48.4	4.0	777.0	56.72
No. of animals per treatment	18,931	12.0	2.0	58.0	9.72
Animal information					
Days in milk (day)	1304	100.4	1.0	323.0	61.15
Parity	1341	1.9	1.0	2.0	0.28
Body weight (kg)	1352	630.3	476.0	861.9	59.35
Dry matter intake (kg/d)	1584	22.1	10.8	32.0	3.73
Milk yield (kg/d)	1584	33.9	10.2	58.5	8.07
Dietary chemical composition (% DM)					
Dry matter (% as-fed)	932	53.3	13.5	96.3	11.62
Organic matter	909	92.4	46.1	98.9	2.63
Crude protein	1510	16.5	9.3	29.6	1.95
Neutral detergent fiber	1392	32.9	21.0	60.8	4.81
Acidic detergent fiber	1103	20.4	10.3	61.6	3.71
Fat	724	3.9	0.3	8.9	1.20
Ash	756	7.3	1.1	16.5	1.56
Non-starch carbohydrate	590	39.5	16.8	51.2	5.30
Starch	872	24.8	0.2	47.6	6.68
Net protein requirements for lactation (g/d)	1584	1014.9	357.9	1923.7	234.26

**Table 3:** Performance of net protein requirement for maintenance (g/d) prediction models developed using random forest regression and support vector regression algorithms, evaluated by test set prediction and 10-fold cross-validation.

	RFR		SVR	
Model	Hold-Out ^1^	10-Fold CV ^2^	Hold-Out	10-Fold CV
R^2^	0.77	0.82	0.65	0.58
RMSEP, g/d	23.35	22.38	29.35	33.34
Mean bias (% RMSEP) **^3^**	0.3	3.4	0.1	2.4
Slope bias (% RMSEP) **^3^**	0.3	1.5	2.8	0.1
Random bias (% RMSEP)	99.4	95.1	97.1	97.5
CCC	0.87	0.89	0.80	0.73

RFR, random forest regression; SVR, support vector regression; NASEM, NRC dairy (2021); RMSEP, root mean square error of prediction; CCC, concordance correlation coefficient. ^1^ Hold-out evaluation: performance on an independent 20% test set (*n* = 57). ^2^ 10-fold cross-validation: cross-validation results on the full dataset (*n* = 1288) using final selected variables. ^3^ There were no statistically significant mean and slope biases (*p* > 0.10).

**Table 4:** Performance of net protein requirement for lactation (g/d) prediction models developed using random forest regression and support vector regression algorithms, evaluated by test set prediction and 10-fold cross-validation.

	RFR		SVR	
Model	Hold-Out ^1^	10-Fold CV ^2^	Hold-Out	10-Fold CV
R^2^	0.79	0.82	0.78	0.76
RMSEP, g/d	90.45	95.17	91.36	109.06
Mean Bias (% RMSEP) **^3^**	0.0	0.4	0.3	0.8
Slope Bias (% RMSEP) **^3^**	0.1	1.2	0.1	1.0
Random Bias (% RMSEP)	99.9	98.4	99.6	98.2
CCC	0.88	0.89	0.88	0.86

RFR, random forest regression; SVR, support vector regression; NASEM, NRC dairy (2021); RMSEP, root mean square error of prediction; CCC, concordance correlation coefficient. ^1^ Hold-out evaluation: performance on an independent 20% test set (*n* = 57). ^2^ 10-fold cross-validation: cross-validation results on the full dataset (*n* = 668) using final selected variables. ^3^ There were no statistically significant mean and slope biases (*p* > 0.10).

## Data Availability

The raw data supporting the conclusions of this article will be made available by the authors upon request. The NANP dataset is available at https://animalnutrition.org/ (accessed on 17 October 2023).
